# The Effect of Saffron Extract Supplementation During Resistance Training on Hippocampal Doublecortin and Hepatic *β*‐Hydroxybutyrate Levels in Rats With Type 2 Diabetes

**DOI:** 10.1155/jdr/1914865

**Published:** 2026-05-15

**Authors:** Vahid Valipour Dehnou, Amin Jalili Sarghaleh, Amin Karimi, Bahman Hasanvand, Hamed Alizadeh Pahlavani

**Affiliations:** ^1^ Department of Sports Sciences, Faculty of Literature and Human Sciences, Lorestan University, Khorramabad, Iran, lu.ac.ir; ^2^ Department of Sports Sciences, Faculty of Literature and Human Sciences, Islamic Azad University, Khorramabad, Iran, azad.ac.ir; ^3^ Department of Physical Education, Farhangian University, Tehran, Iran, cfu.ac.ir

**Keywords:** *β*-hydroxybutyrate, doublecortin, neurogenesis, resistance training, saffron, Type 2 diabetes mellitus

## Abstract

**Background:**

Type 2 diabetes mellitus (T2DM) negatively affects metabolic and neurobiological processes, including increases in hepatic *β*‐hydroxybutyrate (BHB) and reductions in hippocampal doublecortin (DCX). Both saffron extract and resistance training have shown independent benefits in improving glycemic and neuroplastic markers; however, their combined effects on DCX and BHB in diabetic conditions have not been fully examined. This study evaluated the independent and synergistic effects of saffron extract supplementation and resistance training on glucose, hippocampal DCX, and hepatic BHB levels in rats with T2DM.

**Methods:**

Thirty adult male rats were randomly assigned to five groups (*n* = 6 each): control (C), diabetic (D), diabetic with resistance training (DT), diabetic with saffron extract (DS), and diabetic with resistance training plus saffron extract (DTS). Diabetes was induced using streptozotocin (60 mg/kg). Saffron extract (25 mg/kg/day) was administered orally in the DS and DTS groups, and resistance training was performed five sessions per week for 6 weeks. Blood glucose, hippocampal DCX, and hepatic BHB levels were measured. Data were analyzed using one‐way ANOVA with a significance threshold of *p* < 0.05.

**Results:**

Both saffron extract and resistance training independently reduced glucose levels compared with the diabetic group, whereas the combined intervention produced the greatest reduction. DCX levels were significantly higher in the DTS group than in the D, DT, and DS groups (*p* < 0.05). BHB levels in the DT, DS, and DTS groups were significantly lower than in the D group, with the lowest levels observed in the DT and DTS groups (*p* < 0.05).

**Conclusion:**

Saffron extract combined with resistance training more effectively improves glucose regulation, increases hippocampal DCX expression, and reduces hepatic BHB levels in T2DM rats compared with either intervention alone.

## 1. Introduction

Type 2 diabetes mellitus (T2DM) is a prevalent metabolic disorder associated with substantial health and economic burdens worldwide [1, 2]. More than 463 million adults are currently affected, and an additional 374 million individuals live with prediabetes [3]. Beyond its metabolic complications, T2DM is strongly associated with cognitive impairment and dementia, with longitudinal studies indicating nearly a twofold increase in risk [4]. These observations underscore the urgent need to identify interventions that can simultaneously address the metabolic and neurological consequences of diabetes.

Clinically, diabetes is characterized by hyperglycemia, metabolic acidosis, and elevated circulating ketone bodies [5]. Among these, *β*‐hydroxybutyrate (BHB) is the predominant ketone body and shows a marked rise in diabetic ketoacidosis, with the BHB‐to‐acetoacetate ratio increasing up to tenfold. Alterations in hepatic BHB production and reduced hippocampal doublecortin (DCX) expression, a marker of immature neurons, have been documented under diabetic conditions [6].

Disruptions in neurogenesis, synaptic plasticity, and memory pathways render the hippocampus particularly vulnerable to metabolic dysfunction in T2DM [7, 8]. To investigate the metabolic–cognitive consequences of Type 2 diabetes under controlled experimental conditions, rodent models remain indispensable [9]. Accordingly, Sprague–Dawley rats were used, and diabetes was induced using streptozotocin (STZ), a well‐established model that reliably produces chronic hyperglycemia and associated metabolic alterations [10]. Importantly, STZ‐induced diabetic rats exhibit hippocampal dysfunction and cognitive impairments that parallel key features of diabetes‐related cognitive decline observed in human patients [11]. Although this model does not fully recapitulate the progressive insulin resistance characteristic of human Type 2 diabetes, it provides a translationally relevant platform for studying metabolic–cognitive interactions under diabetic conditions [12–14].

DCX plays a central role in neuronal development, and declines in DCX expression contribute to diabetes‐related cognitive impairments. Evidence indicates that exercise enhances hippocampal DCX expression and neurogenesis, thereby counteracting these deficits [15, 16].

During exercise, the liver releases BHB into the circulation. BHB readily crosses the blood–brain barrier and accumulates in the hippocampus, where it induces brain‐derived neurotrophic factor (BDNF) expression through Class I histone deacetylase inhibition [17]. Ketone bodies generated through ketogenic diets also reverse neurogenesis deficits and improve learning and memory by modulating transcriptional pathways via HDAC inhibition [18].

BHB production increases during fasting [19], prolonged exercise, and ketogenic diets, and is elevated in diabetic conditions where it may mitigate cognitive impairments [20]. Acting additionally as a histone dehydrogenase inhibitor, BHB enhances synaptic plasticity [21].

Lifestyle‐based strategies, including exercise, remain central to glycemic regulation in diabetes. Exercise extends lifespan, prevents chronic diseases [22], and improves learning, memory, and cognitive resilience [23]. One mechanism underlying these effects involves increased secretion of FNDC5, a neuroprotective myokine [24]. Saffron aqueous extract has been shown to enhance FNDC5 expression during resistance training [25], and prior research suggests that FNDC5 modulates diabetes‐related outcomes through increased energy expenditure, lipolysis, and insulin sensitivity [26].

Exercise further supports hippocampal neurogenesis through BDNF‐mediated pathways [27–31].

In parallel, herbal interventions have received growing attention for their therapeutic potential in diabetes management [32]. Saffron (*Crocus sativus* L.), traditionally used in China, India, and Iran, possesses antitumor, anti‐inflammatory, antioxidant, and neuroprotective properties and has been applied in the treatment of diabetes and neurodegenerative conditions [1, 33]. Evidence suggests that combining exercise with natural supplements produces greater benefits in diabetic individuals compared with either intervention alone [34]. For example, resistance training combined with vitamin D supplementation improves glycemic indices in T2DM [35] and saffron extract administered during resistance training increases hippocampal BDNF levels in diabetic rats [36]. This combination has also been associated with improvements in memory, antioxidant defenses, and cognitive outcomes [37]. Despite these findings, no study has examined the simultaneous effects of saffron extract and resistance training on hippocampal DCX expression and hepatic BHB levels in T2DM. Therefore, the present study is aimed at investigating the impact of saffron extract administered during resistance training on DCX expression in the hippocampus and BHB levels in the liver of rats with T2DM. We hypothesized that the combined intervention would exert greater benefits than either treatment alone.

## 2. Materials and Methods

### 2.1. Animals

Adult male Sprague–Dawley rats were obtained from the Razi Serum Institute (Shiraz, Iran) and transferred to the animal facility of the Shiraz Stem Cell Center under controlled temperature (22°C ± 2°C) and a 12‐h light/dark cycle. Rats had free access to water and standard chow (Pars Khorakdam Co., Tehran, Iran). An 8‐day acclimatization period was provided before the experiment. All procedures were approved by the Animal Ethics Committee of Lorestan University (LU.ECRA.2017.1).

### 2.2. Induction of Diabetes

Following a 12‐h fast on day eight, diabetes was induced using a single intraperitoneal injection of STZ (50 mg/kg body weight; Sigma, St. Louis, Missouri, United States) freshly dissolved in 0.5 mol/L citrate buffer (pH 4.5) [2]. Control rats received buffer only. Four days later, fasting blood glucose was measured using a Glucotrend 2 glucometer (Roche, Germany). Rats with levels > 300 mg/dL were considered diabetic and enrolled in the study. No animals or data points were excluded during the experiment or subsequent data analysis. In total, 30 rats (308.82 ± 27.57 g) were included.

### 2.3. Experimental Design and Randomization

One week after diabetes induction, rats were randomly assigned into five groups (*n* = 6 each): control (C), diabetic (D), diabetic + resistance training (DT), diabetic + saffron extract (DS), and diabetic + resistance training + saffron extract (DTS). Sample size was determined a priori using G∗Power software (Version 3.1.9.4). For a one‐way ANOVA with five groups, an alpha level of 0.05 and a power of 0.80, a total sample size of 30 animals (*n* = 6 per group) was sufficient to detect a large effect size. Animals were randomly allocated to experimental groups using a computer‐generated randomization sequence. Group allocation was concealed from investigators responsible for outcome assessment and data analysis. All laboratory analyses (ELISA and biochemical assays) were performed by investigators blinded to group allocation. Due to the nature of the interventions, blinding of personnel during supplementation and resistance training was not feasible. To minimize potential confounding factors, standardized housing and experimental procedures were applied throughout the study. All animals were housed under identical conditions (22°C ± 2°C, 12‐h light/dark cycle, ad libitum access to standard chow and water), with four rats per identical cage using the same bedding and environmental enrichment. Animal handling, supplementation, resistance training, and outcome assessments were conducted by the same‐trained personnel using standardized equipment and protocols. To control for cage location and order effects, cages were placed on identical racks and systematically rotated weekly across rack levels and lateral positions according to a predefined schedule. Outcome assessments were performed using a counterbalanced protocol, with the order of group handling, supplementation, training, and measurements rotated daily within the morning assessment window. All standardization, rotation, and counterbalancing procedures were predefined and applied consistently throughout the experimental period.

### 2.4. Animal Welfare, Monitoring, and Humane Endpoints

All experimental procedures were conducted in accordance with the ARRIVE 2.0 guidelines and the principles of the 3Rs (replacement, reduction, and refinement), and were approved by the institutional ethics committee for animal experimentation. Animals were monitored daily throughout the 6‐week intervention for general health status, body weight, food and water intake, locomotor activity, and signs of pain or distress (including abnormal posture, reduced grooming, piloerection, or decreased responsiveness).

Humane endpoints were predefined prior to study initiation. Animals showing severe or persistent signs of distress, injury, or illness, or a body‐weight loss exceeding 15%–20% were to be removed from the study and humanely euthanized. All handling, supplementation, and resistance training procedures were performed by trained personnel to minimize stress, and animals were allowed appropriate acclimatization periods. No unexpected adverse events occurred during the experimental period.

### 2.5. Supplementation (Aqueous Saffron Extract)

A total of 9.2 g of saffron (*C. sativus* L., Iranian Sargol grade; Novin Saffron Co., Iran) was dissolved in 1000 mL of deionized distilled water and incubated at 50°C for 16 h. The solution was filtered and stored at 4°C. The extract was freshly prepared every 72 h to maintain stability. Chemical standardization was confirmed by HPLC/UV/ELSD/MS, demonstrating picocrocin as the major component (20% *w*/*w*), consistent with previous reports. Rats in DS and DTS groups were gavaged once daily with 25 mg/kg body weight of the extract. Supplementation was administered in the morning (9:00–10:00 AM), approximately 4 h before training sessions in combined‐intervention groups [38].

### 2.6. HPLC Analysis

HPLC analysis of the saffron extract was performed using an Agilent 1260 Infinity system equipped with a quaternary pump, an autosampler, a column oven, and a UV–Vis detector. Separation was achieved on a reversed‐phase C18 column (4.6 × 250 mm, 5 *μ*m particle size; Agilent Technologies, United States). The mobile phase consisted of solvent A (water with 0.1% formic acid) and solvent B (acetonitrile). The elution was carried out using a gradient program as follows: 0–5 min, 10% B; 5–20 min, 10%–40% B; 20–25 min, 40%–60% B; 25–30 min, 60%–10% B for re‐equilibration. The flow rate was maintained at 1.0 mL/min, the column temperature was set at 30°C, and the injection volume was 20 *μ*L.

Detection was performed at 350 nm, corresponding to the UV absorption maximum of picocrocin. Identification of compounds was carried out by comparing retention times and UV spectra with reference standards, and confirmation was supported by ELSD and MS detection. Quantification of picocrocin was based on an external calibration curve, and the compound was further structurally verified by NMR analysis. The saffron extract contained 20% picocrocin, consistent with the chromatographic peak area obtained at 350 nm.

### 2.7. Resistance Training Protocol

A 6‐week progressive resistance‐training program was performed using a 1‐m vertical ladder with 2‐cm spacing between rungs. Rats underwent a 1‐week familiarization period, climbing every other day for 3–4 repetitions without added weight.

Training sessions were conducted 5 days per week. At each session, rats completed three warm‐up climbs without load, followed by climbing with weights attached to the base of the tail using leucoplast adhesive (tail tolerance was pretested).

Training commenced at 30% of body weight and progressed to 50%, 75%, 90%, and 100% of the maximum load. For each weight, rats performed two successful repetitions before progressing to the next intensity.

The maximum load was reassessed during the final session of each week by gradually increasing added weight until failure to complete the climb [39].

Standardized training parameters were applied as follows:

• Sets per session: 8–12 climbs (depending on progression).
• Rest interval between climbs: 60–90 s.
• Session duration: approximately 15–20 min.
• Training was not performed to exhaustion; failure tests were limited to weekly load assessment only.

### 2.8. Blood and Tissue Collection

Twenty‐four hours after the final training session, rats were anesthetized with ketamine (40 mg/kg) and xanthine (8 mg/kg). Blood was collected, and hippocampal and liver tissues were dissected and stored at –70°C until analysis [2].

### 2.9. Blood and Tissue Analysis

Serum was obtained by allowing blood to clot for 40 min and centrifuging at 3000 g for 15 min. Glucose levels were analyzed using a commercial glucose oxidase kit.

For hippocampal DCX analysis, 100 mg of tissue was rinsed in 1× PBS, homogenized in 1 mL PBS, frozen at –20°C overnight, subjected to two freeze–thaw cycles, and centrifuged (5000 g, 5 min, 2°C–8°C). Supernatants were analyzed using a commercial ELISA kit (Cusabio DCX; sensitivity 0.078 ng/mL; range 0.312–20 ng/mL; Japan).

For liver BHB, tissue was cut on dry ice and homogenized in 2 mL cold PBS containing protease inhibitor (13 *μ*L/mL; Sigma P8340), diluted to 5 mL, and centrifuged (1500 g, 15 min). The supernatant was aliquoted and stored at –80°C. BHB was measured using an ELISA kit (Cusabio BHB; sensitivity 9.5 nmol/mL; range 50–800 nmol/mL; Japan) [40].

### 2.10. Statistical Analysis

Data were analyzed in SPSS Version 27.0 (SPSS Inc., Chicago, Illinois) and expressed as mean ± SD. Normality was confirmed using the Shapiro–Wilk test (Table 1). One‐way ANOVA followed by Tukey’s post hoc test was used to determine differences between groups. Statistical significance was set at *p* < 0.05.

**Table 1:** Shapiro–Wilk normality test for DCX and BHB across study groups: control (C), diabetes (D), diabetes + training (DT), diabetes + saffron (DS), diabetes + training + saffron (DTS).

	Variable	Group C	Group D	Group DT	Group DS	Group DTS
Shapiro–Wilk test	DCX	0.90207	0.94229	0.94638	0.98821	0.82667
*p* value		0.386	0.678	0.711	0.984	0.101
Shapiro–Wilk test	BHB	0.85225	0.91486	0.86490	0.92712	0.98065
*p* value		0.164	0.469	0.207	0.558	0.955

## 3. Results

**Figure 1:**
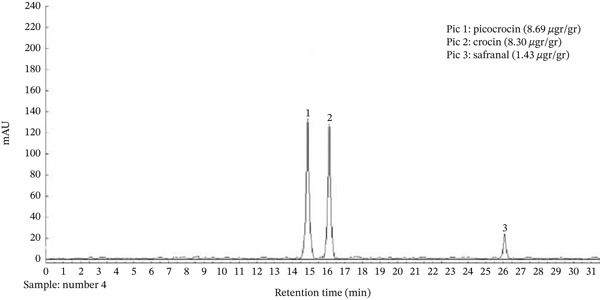
HPLC/UV/ELSD/ESI‐MS chromatogram of saffron extract showing picocrocin as the major compound.

Saffron extract was analyzed using HPLC/UV/ELSD/MS and showed the presence of one major compound, picocrocin (8.69 *μ*g/g) along with other flavonols. The major compound was isolated, and its structure was confirmed by NMR. The amount of this compound in saffron extract was measured by HPLC at UV max (350 nm) and showed the presence of 20.0% in saffron extract (Figure 1).

The results showed a significant difference in blood glucose levels across all groups (*p* < 0.05). Blood glucose levels of all experimental groups were significantly higher than the C group (*p* < 0.05). Blood glucose levels in the DT, DS, and DTS groups were significantly lower than in the D group (*p* < 0.05). (Table 2).

**Table 2:** Blood glucose levels blood glucose levels (mg/dL) in experimental groups. Values are mean ± SD.

Groups	Mean	SD
Group C (control group without diabetes, resistance training, and saffron extract)	97.37	6.18
Group D (diabetes group)	395.2 ^∗^	19.06
Group DT (diabetic group with resistance training)	315.33 ^∗^^#^	20.91
Group DS (diabetic group with saffron extract)	305.44 ^∗^^#^	4.21
Group DTS (diabetic group with resistance training and saffron extract)	291.88 ^∗^^#^	10.69

^∗^Significantly different from the C group (*p* ≤ 0.05). ^#^Significantly different from the D group (*p* ≤ 0.05).

**Figure 2:**
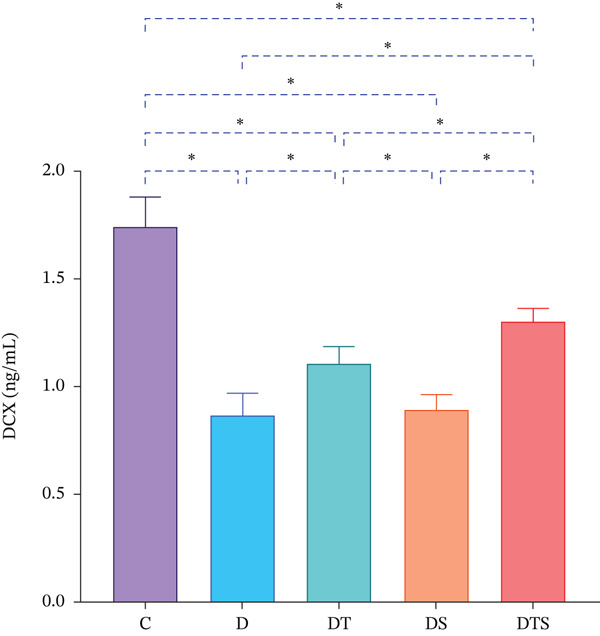
DCX levels. Hippocampal DCX protein levels in control and diabetic groups receiving saffron extract and/or resistance training. Data are presented as mean ± SD. ∗*p* < 0.05.

In addition, the hippocampal DCX protein levels of all experimental groups were significantly lower than the C group (*p* < 0.05). Additionally, Tukey′s post hoc test revealed that hippocampal DCX protein levels of D, DT, and DS groups were significantly lower than the DTS group (*p* < 0.05). However, the hippocampal DCX protein levels of D and DS groups were significantly lower than the DT group (*p* < 0.05) (Figure 2).

**Figure 3:**
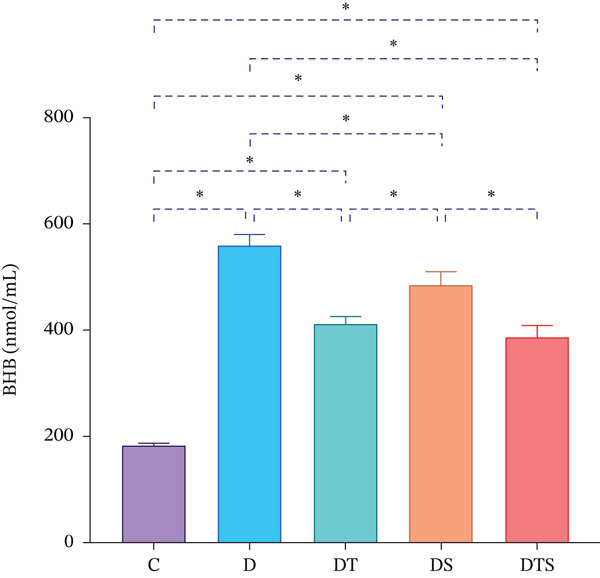
BHB levels. Liver BHB levels in control and experimental groups. Data are presented as mean ± SD. ∗*p* < 0.05.

However, the liver BHB levels of all experimental groups were significantly higher than the C group (*p* < 0.05). Tukey′s post hoc test also showed that the liver BHB levels of DTS, DT, and DS groups were significantly lower than the D group (*p* < 0.05). However, the liver BHB levels of DT and DTS groups were significantly lower than the DS group (*p* < 0.05) (Figure 3).

## 4. Discussion

The present study is aimed at investigating the effect of saffron extract consumption during resistance training on hippocampal DCX expression, hepatic BHB levels, and blood glucose in rats with T2DM. The findings demonstrated that 6 weeks of resistance training combined with saffron extract produced the most favorable outcomes across all measured variables, indicating a potential synergistic effect between these two interventions.

### 4.1. Blood Glucose Levels

In the present study, all diabetic groups showed elevated blood glucose levels compared with the control group, confirming the successful induction of T2DM. Both saffron extract and resistance training significantly reduced blood glucose levels, and the combined intervention (DTS) led to the greatest reduction. These results are consistent with previous findings that physical activity improves metabolic function, reduces the risk of dementia and Alzheimer′s disease, and enhances insulin sensitivity [41]. Saffron extract has also been shown to exert beneficial effects on glycemic indices in individuals with diabetes [42] and in overweight or obese individuals with prediabetes [43]. The glucose‐lowering mechanisms of saffron extract may involve inhibition of renal glucose reabsorption, enhancement of pancreatic beta‐cell regeneration, reduction of insulin resistance, and increased skeletal muscle glucose uptake, largely mediated through AMPK/ACC and MAPK signaling pathways [44–47]. Clinical evidence also supports these findings; for example, a randomized controlled trial reported reductions in inflammatory markers and improvements in fasting glucose and insulin sensitivity after saffron extract supplementation [48].

Resistance training likewise contributes to improved glycemic regulation. Although a few studies have failed to show independent effects of physical activity on HbA1c in T2DM populations [49, 50]. Most evidence supports the beneficial role of resistance training on glycemic markers [51]. Reductions in blood glucose and HbA1c following eccentric, concentric, and combined aerobic–resistance training have been widely documented [52–54]. These findings align closely with the results of the present study, confirming resistance training as an effective nonpharmacological strategy for improving glucose metabolism in diabetes.

### 4.2. BHB Levels

The study also showed that diabetes significantly elevated hepatic BHB levels compared with the control group, whereas saffron extract, resistance training, and especially their combination reduced BHB concentrations. As ketone bodies are produced during states of reduced glucose availability, their elevation is a hallmark of diabetes and contributes to the pathophysiology of DKA [52]. Although ketone bodies, particularly BHB, serve as alternative energy substrates and can exert neuroprotective effects—such as in epileptic conditions [55]. Their chronic elevation reflects impaired glucose utilization.

Exercise can modulate ketone metabolism, partly by increasing BDNF levels and enhancing insulin sensitivity [17, 56]. Mechanistically, BHB can cross the blood‐brain barrier and induce BDNF expression while acting as an epigenetic regulator via inhibition of Class I HDACs [57, 58]. In diabetes, increased hepatic BHB may function as a compensatory response to declining BDNF levels [59, 60].

The reductions in BHB observed in this study following saffron extract and/or resistance training indicate improved glucose metabolism and reduced reliance on ketogenesis. This mechanism may also attenuate the risk of DKA by shifting energy production away from ketone bodies toward glucose oxidation [61] Additionally, saffron extract has demonstrated cognitive benefits beyond metabolic regulation, including improvements in mood, memory, and increases in hippocampal BDNF levels [62, 63]. Similar findings have been reported for resistance training and combined interventions [36].

### 4.3. DCX Protein Levels

DCX is a critical marker of neurogenesis, and reduced expression is associated with impaired hippocampal plasticity, learning, and memory deficits [64–66]. Exercise has been shown to increase DCX levels [67], although not consistently across all models of physical activity [68]. One potential pathway involves cathepsin B, an exercise‐induced myokine that enhances hippocampal neurogenesis and DCX expression [69]. Other natural supplements, such as calcium and vitamin D, have also been reported to increase hippocampal DCX levels [70].

The results of the present study demonstrated that resistance training significantly increased DCX expression, and the combination of resistance training with saffron extract produced the highest levels. In contrast, saffron extract alone did not significantly increase DCX compared with the diabetic group. The observed increase in DCX levels may contribute to improved hippocampal function and memory [71] consistent with the neuroprotective and anti‐inflammatory properties of saffron′s active constituents [72].

In the present study, saffron extract supplementation alone did not result in a significant change in hippocampal DCX levels. Nevertheless, previous studies have reported that saffron and its bioactive constituents may modulate multiple neurobiological pathways, including acetylcholinesterase inhibition, dopaminergic and amyloid‐*β* signaling, attenuation of oxidative stress, regulation of microglial activation, and modulation of mitochondrial function and the Nrf2 pathway [73]. In addition, increases in neurogenesis‐ and plasticity‐related markers such as DCX, BDNF, NeuN, and Sox2 have been observed following saffron administration in other experimental models [74], The lack of a significant effect of saffron supplementation alone in the current study suggests that these neurobiological actions may depend on the activation of complementary signaling pathways [75, 76]. Resistance training is known to enhance hippocampal neuroplasticity through mechanisms such as increased BDNF availability, improved metabolic control, and reduced oxidative stress [77]. Therefore, the observed enhancement of DCX expression in the combined training and saffron group (DTS) may reflect a synergistic interaction, whereby resistance training provides a permissive neurobiological environment that enables saffron extract to potentiate exercise‐induced neurogenesis [28, 78].

Reinforcing its potential in enhancing neurogenesis. The combination of exercise and saffron supplementation may therefore produce additive or synergistic benefits that enhance neural plasticity and counteract diabetes‐related cognitive decline [79]. These findings are supported by Valipour et al., who observed increased BDNF expression with saffron extract during resistance training in diabetic rats [36].

## 5. Strengths and Limitations

The use of an animal model limits generalizability to humans. Although increased hippocampal DCX suggests enhanced neurogenesis, the absence of behavioral assessments precludes conclusions regarding functional recovery; thus, future studies should include behavioral testing, varied saffron doses, alternative exercise modalities, longer interventions, and clinical populations.

A major strength of this study is the combined use of resistance training, a well‐established metabolic intervention, and saffron extract, a safe natural supplement with reported neuroprotective effects, allowing concurrent targeting of metabolic and cognitive impairments in T2DM.

Interpretation should also consider the STZ model, which primarily induces *β*‐cell damage and does not fully represent insulin resistance. Nevertheless, it remains a valid model for studying hyperglycemia‐related metabolic and neurobiological alterations; therefore, the present findings mainly reflect the effects of resistance training and saffron extract under sustained hyperglycemia. Use of alternative T2DM models may improve translational relevance.

## 6. Conclusion

This study indicates that resistance training serves as the primary stimulus for improving glucose metabolism, BHB, and DCX expression in rats with T2DM, whereas saffron extract supplementation exerts a synergistic, potentiating effect on training‐induced adaptations. Although the combined intervention produced greater improvements than either intervention alone, the observed changes in DCX and BHB were predominantly driven by resistance training, with saffron extract acting as a supportive modulator rather than an independent determinant. Future studies employing different saffron dosages, alternative exercise modalities, longer intervention periods, and human populations are warranted.

## Author Contributions

Vahid Valipour Dehnou and Bahman Hasanvand: designed the research study. Amin Jalili Sarghaleh and Amin Karimi: performed the research. Vahid Valipour Dehnou and Hamed Alizadeh Pahlavani: provided help on investigation and methodology. Vahid Valipour Dehnou and Hamed Alizadeh Pahlavani: analyzed the data. Vahid Valipour Dehnou, Bahman Hasanvand, Amin Karimi, and Amin Jalili Sarghaleh: wrote the manuscript. All authors contributed to editorial changes in the manuscript. All authors have participated sufficiently in the work.

## Funding

No funding was received for this manuscript.

## Disclosure

All authors read and approved the final manuscript. All authors have agreed to be accountable for all aspects of the work.

## Ethics Statement

All experiments in the current study followed the ethical principles approved by the Animal Ethics Committee of Lorestan University (Reference Number: LU. ECRA. 2017.1).

## Conflicts of Interest

The authors declare no conflicts of interest.

## Data Availability

Data are available on request from the authors.
